# Identifying New Hybrid Insulin Peptides (HIPs) in Type 1 Diabetes

**DOI:** 10.3389/fimmu.2021.667870

**Published:** 2021-04-30

**Authors:** Stuart I. Mannering, Alan F. Rubin, Ruike Wang, Pushpak Bhattacharjee

**Affiliations:** ^1^ Immunology and Diabetes Unit, St. Vincent’s Institute of Medical Research, Melbourne, VIC, Australia; ^2^ Department of Medicine, University of Melbourne, St. Vincent’s Hospital, Melbourne, VIC, Australia; ^3^ Bioinformatics Division, Walter and Eliza Hall Institute of Medical Research, Melbourne, VIC, Australia; ^4^ Department of Medical Biology, University of Melbourne, Melbourne, VIC, Australia

**Keywords:** hybrid insulin peptides (HIPs), CD4^+^ T cell, autoimmunity, type 1 diabetes, epitope

## Abstract

In 2016 Delong et al. discovered a new type of neoepitope formed by the fusion of two unrelated peptide fragments. Remarkably these neoepitopes, called hybrid insulin peptides, or HIPs, are recognized by pathogenic CD4^+^ T cells in the NOD mouse and human pancreatic islet-infiltrating T cells in people with type 1 diabetes. Current data implicates CD4^+^ T-cell responses to HIPs in the immune pathogenesis of human T1D. Because of their role in the immune pathogenesis of human T1D it is important to identify new HIPs that are recognized by CD4^+^ T cells in people at risk of, or with, T1D. A detailed knowledge of T1D-associated HIPs will allow HIPs to be used in assays to monitor changes in T cell mediated beta-cell autoimmunity. They will also provide new targets for antigen-specific therapies for T1D. However, because HIPs are formed by the fusion of two unrelated peptides there are an enormous number of potential HIPs which makes it technically challenging to identify them. Here we review the discovery of HIPs, how they form and discuss approaches to identifying new HIPs relevant to the immune pathogenesis of human type 1 diabetes.

## Introduction

Type 1 diabetes (T1D) is an autoimmune disease caused the T-cell mediated destruction of the pancreatic beta cells (1). How and why the immune system turns against otherwise healthy tissue, to cause an autoimmune disease, is currently not known. However, for T1D it is clear that T-cells infiltrate the islet of Langerhans within the pancreas and destroy the insulin producing beta cells (2).

Genetic studies have revealed that risk of developing T1D is associated with class II HLA (3). The strongest genetic risk for developing T1D is associated with the HLA class II haplotypes HLA-DR3-DQ2 (Odds ratio (OR)>3.6) and HLA-DR4-DQ8 (OR>11.37) (3). Within these haplotypes the HLA-DQ alleles confer the majority of the risk (4). Intriguingly, people who are HLA-DR3-DQ2; DR4-DQ8 heterozygous are at greatest risk (OR>16.6) of developing type 1 diabetes (3). HLA-DQ2/8 heterozygous antigen presenting cells express four types of HLA-DQαβ heterodimers. The ‘regular’ HLA-DQ2*cis* and –DQ8*cis* (α and β chains encoded on the same chromosome) and the ‘*trans*’ dimers (α and β chains encoded on different chromosomes) (5). Collectively, this work suggests that CD4^+^ T-cell responses against beta cell derived antigens play a central role in the pathogenesis of T1D, the identity of the antigens targeted by pathogenic CD4^+^ T cells has not been fully resolved.

Insulin and its precursor, proinsulin, have long been considered to be central to the autoimmune pathogenesis of T1D (1, 6). Proinsulin is the major protein product of beta cells accounting for ~10% of the cell’s protein (7). Mature insulin forms when the central ‘C-peptide’ of proinsulin is excised in the beta-cell granules leaving mature insulin and ‘free’ C-peptide (8). A genetic susceptibility locus maps to a variable number of tandem repeats (VNTR) upstream of the insulin gene and regulates its expression in the thymus, which in turn affects central tolerance to (pro)insulin (9). This locus is strongly associated with risk of developing type 1 diabetes (OR>2.5), second only to the HLA (10). In 2015, Pathiraja et al. (11) were the first to show that proinsulin specific CD4^+^ T cells infiltrate human islets in type 1 diabetes. Human islet-infiltrating CD4^+^ T cells recognize several epitopes derived from the C-peptide presented by HLA-DQ8, or DQ8*trans* (11).

## The Discovery of Hybrid Insulin Peptides (HIPs)

An important twist in proinsulin’s role in T1D came in 2016 when Delong et al. (12) described hybrid insulin peptides (HIPs). A hybrid insulin peptide, or HIP, is a CD4^+^ T-cell epitope that is formed by the posttranslational fusion of two peptide fragments [reviewed by (13)]. To date, as the name suggests, at least one of the peptide fragments derives from insulin or proinsulin. The peptide fragments that form a HIP can be derived from the same parental protein molecule(s), or from two distinct proteins. Delong and Haskins’ goal was to identify the epitopes recognized by a family of T-cell clones, known as BDC, that had been isolated from NOD mice (14). Importantly, many of the BDC lines were known to cause diabetes (15). Because of their clear pathogenic role, the BDC clones have been well studied, particularly BDC-2.5 (16, 17). However, the naturally arising agonist epitope for BDC-2.5, and many other BDC clones, remained elusive. Several mimotopes peptides were identified that stimulated BDC-2.5 (18–20), but a broadly accepted, naturally arising, target antigen was not found. Despite this, it became clear that an epitope from the beta-cell secretory granule protein, chromogranin-A (ChgA) was, at least, a partial target (21, 22). More specifically, it was clear that the WE14 fragment of chromogranin-A could stimulate the BDC-2.5 cells albeit weakly. Synthetic peptide analogues of the WE14 fragment were made with amino acid substitutions at each position and tested for their capacity to stimulate BDC-2.5 cells. This analysis showed that substitutions at the carboxyl terminus of the putative epitope impacted upon BDC-2.5 stimulation (22). Furthermore, a membrane extract from a beta-cell tumor was a more potent stimulator of BDC-2.5 than synthetic WE14 peptide (22) - clearly something was missing.

Delong, Haskins and co-workers resolved the enigma of the natural agonist for BDC-2.5 (and several other BDC lines) when they made the intuitive leap that CD4^+^ T-cell epitopes could form by fusion of two peptide fragments. They showed, by mass spectrometry and functional T-cell assays, that the missing half of the epitope was a fragment of the C-peptide from proinsulin (LQTLAL). The optimal agonist for BDC-2.5 (and BDC-9.46 and BDC-10.1) was a peptide formed by the fusion of a fragment of C-peptide with a fragment derived from WE14 (LQTLAL-WSRMD). A similar HIP (LQTLAL-NAARD) formed by the same C-peptide fragment (LQTLAL) fused to a fragment of IAPP2 (NAARD) was also identified. These new types of peptides were called ‘hybrid insulin peptides’ or HIP. Titration experiments, using synthetic peptides, revealed that a Cpeptide-ChgA HIP was an extremely potent (at nM concentrations) stimulator of BDC-2.5 CD4^+^ T cells, making it a very plausible candidate for the natural agonist for BDC-2.5. Solving the mystery of the ligand for BDC-2.5 was interesting, but it was not clear if HIP specific CD4^+^ T cells arose in people with T1D and if they played any role the pathogenesis of T1D. We addressed this question by testing a panel of 16 putative human HIPs, designed based on the HIPs identified in the NOD mouse, against of human islet-infiltrating CD4^+^ T-cell clones. From these experiments we identified two clones that responded to a Cpeptide-IAPP2 HIP (12). Independently, Sally Kent’s group screened the same panel of 16 HIPs and identified an islet-infiltrating CD4^+^ T-cell line that responded to a Cpept-NYP HIP (12). Subsequently, further evidence for a pathogenic role of CD4^+^ T-cell responses to HIPs has accumulated. For example, further screening of this panel of 16 HIPs against human islet-infiltrating T-cells lines revealed responses to Cpept-IAPP1, Cpept-InsA and Cpept-IAPP2 HIPs (23) (See **Table 1**). Analysis of HIP specific responses in the PBMC of people with recent onset T1D revealed responses to several HIPs that were less frequently detected in control subjects who did not have T1D (25).

**Table 1:** Summary of currently known HIPs.

Proteins	HIP amino acid sequence	HLA	Origin of T cells	Ref
Cpept-IAPP2	GQVELGGG-NAVEVLK	DQ8	Islet-infiltrating T cells	(12)
Cpept-Neuropept Y	GQVELGGG-SSPETLI	ND	Islet-infiltrating T cells	(12)
Cpept-InsA	GQVELGGG-GIVEQCC	ND	Islet-infiltrating T cells	(23)
Cpept-IAPP1	GQVELGGG-TPIESHQ	ND	Islet-infiltrating T cells	(23)
Cpept-IAPP2	GQVELGGG-NAVEVLK	ND	Islet-infiltrating T cells	(23)
InsB-SG1	VCGERGFF-EELVARSE	DR4	PBMC	(24)
InsB-SG1	HLVEALYL-EELVARSE	DR4	PBMC	(24)
InsA-InsB	ICSLYQLE-FVNQHLCG	DR4	PBMC	(24)
Cpept/InsA-SG1	SLQKRGIV-EELVARSE	DR4	PBMC	(24)
InsA-SGV	CSLYQLEN-SVPHFSDE	DR4	PBMC	(24)
Cpept-GRP78	QPLALEGS-ALSSQHQA	DR4	PBMC	(24)

## How do HIPs Form?

Protease-mediated peptide splicing, or transpeptidation, has been described in bacteria (26), plants (27), *in vitro* (28) and in humans. In humans, proteasome-mediated protein splicing generates epitopes recognized by tumor specific CD8^+^ T cells (29, 30). In all cases energy released during proteolysis is ‘recycled’ to drive the formation of a peptide bond (30). Under most circumstances this ‘reverse proteolysis’ is very inefficient, but high protein concentrations in a confined environment favor protease mediated peptide fusion (31). Beta-cell granules are extremely densely packed with insulin, C-peptide and several other proteins including chromogranins and amyloid proteins (32). Furthermore, the beta-cell granules are the site of very active proteolysis. Many proteins found in the beta cell granules, including proinsulin (PI), chromogranins (Chg) and islet amyloid polypeptide (IAPP), are cleaved by the granule proteases to generate an array of bio-active peptides (32). However, Wan et al. (33) reported that analysis of mass spectrometry data from beta-cell crinosomes revealed the presence of HIPs suggesting that HIPs form in the crinosomes. More recently, Reed et al. (34) reported that when the appropriate peptides are digested by the lysosomal protease, cathepsin L, HIPs that activate the lines BDC-2.5 and BDC-6.9 are generated. This led to the suggestion that HIPs form when senescent insulin granules fuse with lysosomes to form crinophagic granules. The high concentration of insulin granule proteins and cathepsin-L favors the formation of HIPs. Crinophagic granules can be taken up by antigen presenting cells allowing the HIPs to be presented to CD4^+^ T cells (35). Nonetheless, the precise location(s) of HIP formation remain unclear. C-peptide can be cleaved at sites other than the dibasic residues at the B-C and C-A chain junctions (36). This indicates that other proteases may mediate the formation of HIPs. It also remains possible that transpeptidation and HIP formation occurs in both granules and crinosomes.

## Identifying New HIPs

**Figure 1:**
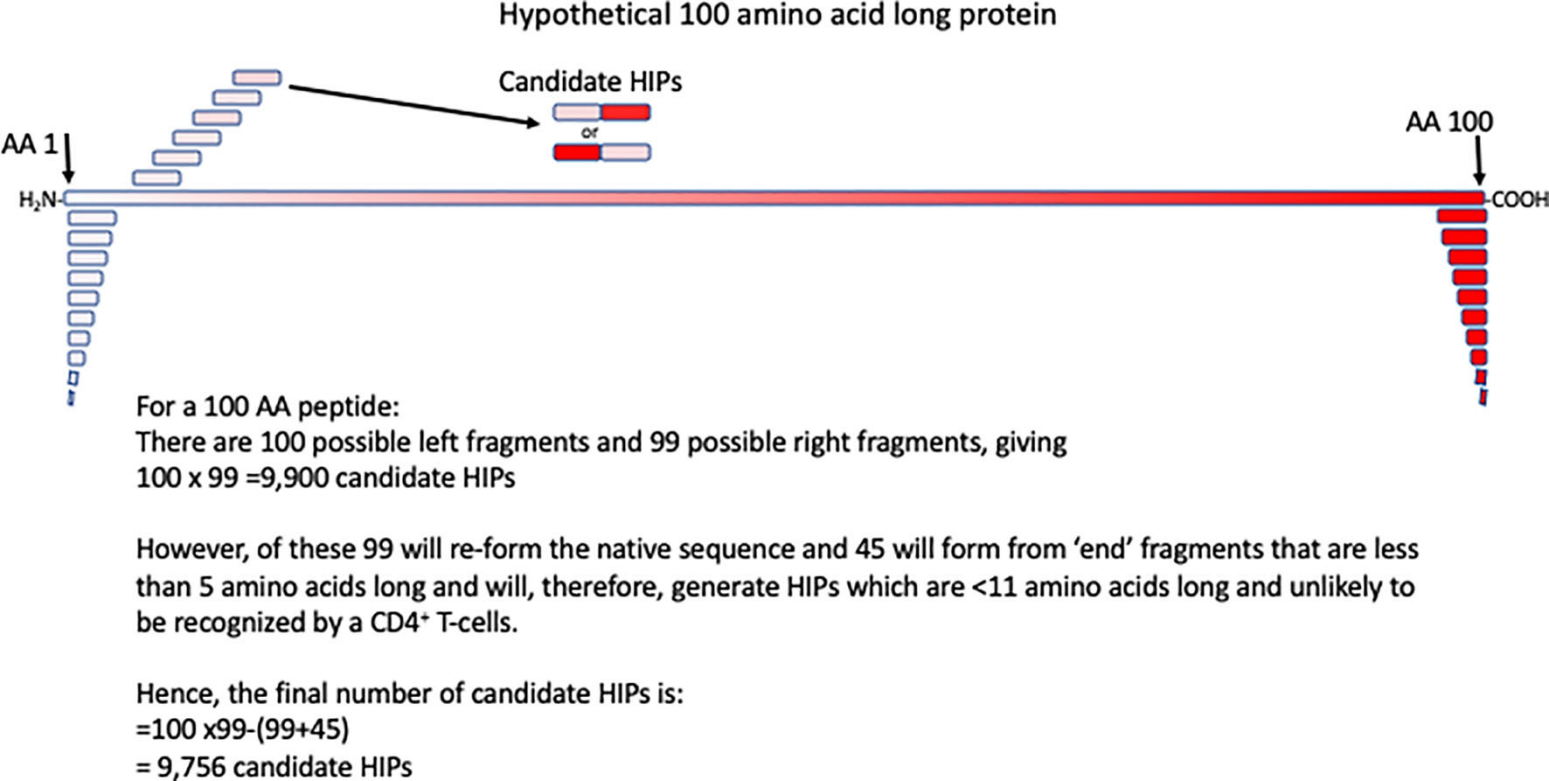
Calculating the number of HIPs that could be generated from a 100 amino acid (AA) long protein. A hypothetical 100 amino acid long peptide is indicated by the red box. The first few of all possible 10 amino acid fragments, derived from this protein are shown in red. Potential HIPs, in both orientations of the composite fragments are shown. Assumptions made in calculating the number of potential HIPs that may form from a 100 amino acid protein are shown.

Identification of ‘regular’ T-cell epitopes has been scientific challenge, but over the past decades significant progress has been made (37). Progress has been facilitated by advances in peptide synthesis techniques, T-cell cloning (38), TCR expression, recombinant DNA techniques and mass spectrometry. Mapping of ‘regular’ T-cell epitopes usually reduces the target epitope from a cell, or microbe, to a protein and from there an ~8-14 amino acid peptide. However, in the case of HIPs the process is more complex, because, theoretically at least, any protein fragment can fuse with any other protein fragment. HIPs can form from fragments of the same proteins, or from two different proteins, and the fragments can fuse in either order. These possible combinations give rise to an enormous array of HIPs that are, theoretically at least, possible. For example, from a protein of 100 amino acids we calculate that there are 9,756 possible HIPs (**Figure 1**). This only included possible HIPs formed by fragmentation and fusion of a single 100 amino acid protein. Once other candidate proteins are considered as potential HIP fragment donors the number of possible HIPs becomes extremely large. Below we discuss the different approaches to identifying and validating novel HIPs at scale.

Mass spectrometry has been invaluable for the identification of HIPs. The advantage of mass spectrometry is that a large number of sequences can be identified rapidly. Candidate T cell epitopes have been identified by elution of peptides from HLA/MHC on the surface of antigen-presenting cells. However, this approach is not feasible with HIPs which are present at very low concentrations in beta cells. Furthermore, since HIPs aren’t directly encoded new algorithms have been developed to facilitate the identification of HIPs, or CD8^+^ T-cell epitopes formed by proteosome mediated peptide splicing (39, 40). Nonetheless, a rigorous mass spectrometry work flow for the identification of HIPs from beta-cell extracts has been developed (41).

One of the disadvantages of mass spectrometry is large numbers of cells are usually required to isolate sufficient material for analysis. This makes the analysis of primary beta cells difficult. Delong et al. solved this problem by using beta cell tumors from RIP-TAg mice. More recently they have adapted their work-flow to allow for smaller numbers of cells (41) and developed internal controls to allow quantifiable confidence when matching synthetic and biologically derived peptides (42). Mass spectrometry analysis provides good evidence for the presence of a peptide, but it does not give any insights into the capacity of T cells to respond to a particular peptide for their relationship between a CD4^+^ T-cell response to an epitope and the disease pathogenesis. For this reason, validation of candidate HIPs with functional T-cell assays, as described above, is essential.

Arribas-Layton et al. (24) used a tetramer-based approach to identify new HIPs. They started with a database of 7,654 candidate HIPs formed by fusion of fragments of proinsulin with a variety of beta cell granule proteins. This list of peptides was stratified by predicted binding to HLA-DR4 (DRB1*04:01). The 50 peptides with the strongest predicted binding affinity to HLA-DR4 were then screened in competitive HLA binding assays and 30 confirmed HLA-DR4 binding peptides were chosen. These 30 HIPs were used to make HLA-DR4/HIP tetramers. Finally, PBMC from individuals with T1D were stimulated with pools of HIP peptides and cytokines for two weeks, then the expanded cells were stained with DR4/HIP tetramers. This work led to the identification of six new, HLA-DR4 restricted HIPs (**Table 1**).

## Discussion

The work summarized above highlights the power of combining functional T-cell responses with proteomic analysis. Observations using T-cell responses support the proteomics data and, in turn, the proteomics supports the immunology. This work brings us to the current question: is it possible to develop a high throughput and efficient protocol for identifying HIPs that are relevant to the immune pathogenesis of human T1D? If so, what would such a protocol look like? The primary challenge, as outlined above, is the enormous number of potential HIPs to be screened. Proteomic approaches use fractionation of tissue lysates and screening with disease-relevant T cells. While this approach worked for Delong et al. (12), who had the BDC lines and RIP-Tag beta cells, it will be challenging to apply this to human studies because sufficiently large numbers of beta cells are not readily available and CD4^+^ T cells of clear clinical relevance are not available. CD4^+^ T cells that infiltrate human islets (11, 43) are available, but screening large numbers of candidate HIPs against large numbers of islet-infiltrating T cells remains a challenging task.

The development of improved synthetic peptide and DNA technologies increases the feasibility of screening approaches. Large libraries of candidate HIPs can be generated *in silico* and as peptides or synthetic DNA. For example, Arribas-Layton et al. refined their pool of over 7,000 candidates HIPs by predicting and then confirming binding to HLA-DR4. This gave them a manageable number of HIPs to screen with PBMC from people with T1D using DR4/HIP tetramers. The limitation of this approach is that it focuses on a single HLA restriction and relies upon the accuracy of the HLA-binding predictions.

An ideal HIP-identification protocol would have the following features: (i) it would allow the screening of very large numbers of candidate HIPs, reducing the incentive to use HLA binding predictions to enrich for candidates with higher predicted binding capacities. (ii) it would screen full haplotypes of T1D-associated HLA alleles at one time, avoiding the need to screening for a particular restriction. (iii) it would identify epitopes based on their capacity to stimulate disease relevant T cells, such as CD4^+^ T cells isolated from the islets of organ donors who had T1D. The frequency of beta cell antigen specific T cells in peripheral blood is too low to allow these cells to be used in screening assays. Expansion of cells of possible relevance biases the screen towards the specificities already thought to be important. To our knowledge, an assay that meets all these criteria does not currently exist. Recent developments may make such an assay feasible. For example, the T-Scan protocol (44), which can screen large numbers of candidate CD8^+^ T-cell epitopes, has many of the desirable feature. Our challenge now is to develop a comparable assay that can be used to identify human CD4^+^ T-cell epitopes, including HIPs.

The discovery of HIP peptides has revealed a new array of targets for the autoimmune responses underlying type 1 diabetes. Discoveries made using the NOD mouse have rapidly been supported by experiments using human materials. It is now clear that HIPs do form in beta cells and can be the targets of autoimmune responses. However, it seems very likely that we have only identified a fraction of the HIPs that may play a role in T1D. Emerging approaches to identify and validate new HIPs. Once defined these epitopes will be of great interest, and utility, in T cell assays to monitor changes in beta cell autoimmunity (45) and antigen-specific therapies for T1D.

## Author Contributions

SM and PB conceived the scope and focus of the review. SM wrote the first draft. AR, RW and PB contributed to the editing and preparation of the final manuscript. All authors contributed to the article and approved the submitted version.

## Funding

This work was supported by the Juvenile Diabetes Research Foundation (2-SRA-2020-909-S-B) and the National Health and Medical Research Council (NHMRC GNT123586, 1138717) (SM). PB is supported by a St. Vincent’s Institute Rising Star Award. The authors acknowledge the support of the Operational Infrastructure Support Program of the Victorian Government.

## Conflict of Interest

The authors declare that the research was conducted in the absence of any commercial or financial relationships that could be construed as a potential conflict of interest.
